# Overexpression of IκBα in cardiomyocytes alleviates hydrogen peroxide-induced apoptosis and autophagy by inhibiting NF-κB activation

**DOI:** 10.1186/s12944-020-01327-2

**Published:** 2020-06-24

**Authors:** Min Han, Xiao-Cui Chen, Ming-Hui Sun, Min-Tao Gai, Yi-Ning Yang, Xiao-Ming Gao, Xiang Ma, Bang-Dang Chen, Yi-Tong Ma

**Affiliations:** 1 Xinjiang Key Laboratory of Cardiovascular Disease Research, Clinical Medical Research Institute, the First Affiliated Hospital of Xinjiang Medical University, Urumqi, 830054 PR China; 2 Department of Cardiology, the First Affiliated Hospital of Xinjiang Medical University, Urumqi, 830054 PR China; 3 Department of Nephrology, Fifth Affiliated Hospital of Xinjiang Medical University, Urumqi, 830000 PR China

**Keywords:** Nuclear factor kappa B, Inhibitor of kappa B alpha, Apoptosis, Autophagy, Adeno-associated virus serotype 9, Oxidative stress, Cardiomyocytes

## Abstract

**Background:**

Inflammation and oxidative stress play predominant roles in the initiation and progression of ischaemia/reperfusion (I/R) injury, with nuclear factor kappa B (NF-κB) serving as a crucial mediator. Overexpression of the inhibitor of κB alpha (IκBα) gene is hypothesized to have protective effects against apoptosis and autophagy in cardiomyocytes subjected to hydrogen peroxide (H_2_O_2_) by inhibiting the NF-κB pathway.

**Methods:**

The IκBα^S32A, S36A^ gene was transfected via adeno-associated virus serotype 9 (AAV9) delivery into neonatal rat ventricular cardiomyocytes (NRVMs) prior to H_2_O_2_ treatment. NRVMs were divided into control, H_2_O_2_, GFP + H_2_O_2_, IκBα+H_2_O_2_, and pyrrolidine dithiocarbamate (PDTC) + H_2_O_2_ groups. Nuclear translocation of the NF-κB p65 subunit was evaluated by immunofluorescence and Western blotting. Cell viability was assessed by Cell Counting Kit-8 assay. Supernatant lactate dehydrogenase (LDH) and intracellular malondialdehyde (MDA) were measured to identify H_2_O_2_-stimulated cytotoxicity. Apoptosis was determined by Annexin V-PE/7-AAD staining, and the mitochondrial membrane potential (ΔΨm) was detected by JC-1 staining. Western blotting was used to detect apoptosis- and autophagy-related proteins.

**Results:**

IκBα transfection significantly increased cell viability and ΔΨm but decreased the supernatant LDH and cellular MDA levels in cardiomyocytes exposed to H_2_O_2_. Meanwhile, IκBα overexpression decreased H_2_O_2_-induced apoptosis by upregulating the Bcl-2/Bax ratio and reduced autophagy by downregulating the expression of Beclin-1 and the LC3-II/LC3-I ratio. These effects partly accounted for the ability of IκBα to inhibit the NF-κB signalling pathway, as evidenced by decreases in p65 phosphorylation and nuclear translocation. Indeed, the effects of inactivation of NF-κB signalling with the specific inhibitor PDTC resembled the cardioprotective effects of IκBα during H_2_O_2_ stimulation.

**Conclusion:**

IκBα overexpression can ameliorate H_2_O_2_-induced apoptosis, autophagy, oxidative injury, and ΔΨm loss through inhibition of the NF-κB signalling pathway. These findings suggest that IκBα transfection can result in successful resistance to oxidative stress-induced damage by inhibiting NF-κB activation, which may provide a potential therapeutic target for the prevention of myocardial I/R injury.

## Introduction

Acute myocardial infarction (AMI) is the leading cause of death worldwide, and reperfusion therapy is the most effective treatment for AMI [1]. Paradoxically, the process of myocardial reperfusion also induces a series of adverse cardiac events such as inflammation, necrosis, apoptosis and autophagy, ultimately leading to myocardial ischaemia/reperfusion (I/R) injury [2]. Recent evidence has suggested that excessive inflammation and oxidative stress play predominant roles in the initiation and progression of I/R injury [3, 4].

Nuclear factor kappa B (NF-κB) is an inflammatory inducer and redox-sensitive transcription factor in most cell types [5]. The p65/50 heterodimer, the most common pattern of NF-κB dimer, normally exists as a component of inactive cytoplasmic complexes bound to the inhibitor of κB alpha (IκBα). Upon stimulation, IκBα is phosphorylated and undergoes ubiquitylation and proteasomal degradation, subsequently leading to phosphorylation and nuclear translocation of the NF -κB p65 subunit [6]. Activated NF-κB then initiates the expression of corresponding target genes, many of which may regulate apoptosis, inflammation and autophagy [7].

However, whether NF-κB is protective or detrimental for cardiomyocyte apoptosis remains controversial [8]. Notably, our previous study indicated that the p65 ribozyme could prevent cell apoptosis in H9C2 cardiomyocytes exposed to hydrogen peroxide (H_2_O_2_) [9]. Autophagy, an evolutionarily conserved form of “self-digestion”, plays dual roles in the heart [10]. Recent studies on autophagy have shown both the protective [11] and deleterious [12] effects of autophagy in cardiomyocytes against oxidative stress. Evidence has revealed a strong correlation between modulation of NF-κB and the autophagic response [13, 14]. In addition, cross-talk between autophagy and apoptosis has been noted [15], and NF-κB is known to mediate the balance between autophagy and apoptosis [16].

Therefore, NF-κB activation is thought to be the key point of I/R injury; thus, inhibiting NF-κB may be a targeted therapy for I/R injury. Phosphorylation of IκBα, the key inhibitor of the canonical NF-κB pathway, at Ser 32 and Ser 36 is necessary for its degradation, and any mutation of these two serine residues blocks IκBα degradation [6]. Recently, adeno-associated virus serotype 9 (AAV9) was demonstrated to be the best gene carrier due to its high efficiency in the heart [17]. H_2_O_2_, a common reactive oxygen species (ROS), is generally utilized to mimic I/R injury in vitro [12]. Thus, the IκBα^S32A, S36A^ gene was transfected into cardiomyocytes via AAV9-mediated delivery to investigate the role of inhibition of the NF-κB pathway in H_2_O_2_-induced apoptosis and autophagy. Pyrrolidine dithiocarbamate (PDTC), a specific inhibitor of NF-κB, was used as a positive control in this study.

## Materials and methods

### Ethics statement

The experimental protocol was approved by the Ethics Committee of the First Affiliated Hospital of Xinjiang Medical University (No. IACUC-20180223-69). One- to three-day-old neonatal Sprague-Dawley (SD) rats were purchased from the Experimental Animal Center of Xinjiang Medical University and handled in accordance with the recommendations in the Guidelines for the Care and Use of Laboratory Animals of the National Institutes of Health.

### Reagents

Briefly, rabbit anti-Bax polyclonal antibody (#2772) and rabbit anti-p65 (#8242), anti-p-p65 (#3033), anti-IκBα (#4812), anti-GFP (#2956), anti-Beclin-1 (#3495), and anti-LC3 II/I (#12741) monoclonal antibodies were all obtained from Cell Signalling Technology (Danvers, MA, USA). Rabbit anti-Bcl-2 (ab196495), anti-Histone H3 (ab1791) and anti-β-actin (ab8227) polyclonal antibodies and horseradish peroxidase (HRP)-conjugated anti-rabbit secondary antibody (ab205718) were obtained from Abcam (Cambridge, UK). RIPA buffer and Halt™ Protease and Phosphatase Inhibitor Cocktail were obtained from Thermo Fisher Scientific (Waltham, MA, USA). Enhanced chemiluminescence (ECL) reagent and JC-1 were obtained from Millipore (Bedford, MA, USA). Trypsin, PDTC and bromodeoxyuridine (BrdU) were obtained from Sigma (St. Louis, MO, USA). Dulbecco’s modified Eagle’s medium (DMEM), foetal bovine serum (FBS), and penicillin-streptomycin solution were obtained from Gibco (Grand Island, NY, USA). Collagenase II was obtained from Worthington (Minnesota, USA). H_2_O_2_ was obtained from Sangon (Shanghai, China).

### Vector design

Recombinant AAV-9 vectors generated by a recombinant baculovirus (rBac)-based system in SF9 cells as previously described were purchased from Virovek (Hayward, CA, USA) [18]. The recombinant AAV9 vectors were packaged as double-stranded DNA and contained the enhanced green fluorescent protein (eGFP) gene (dsAAV9-GFP) or the IκBα^S32A, S36A^ gene (dsAAV9-IκBα) driven by the human cytomegalovirus (CMV) promoter.

### Isolation and culture of rat cardiomyocytes

The protocol for the isolation and purification of neonatal rat ventricular cardiomyocytes (NRVMs) was reported in our previous study [19]. Briefly, the hearts of 1- to 3-day-old neonatal SD rats were dissected and digested with 0.1% trypsin and 0.08% collagenase II. Following differential adhesion twice for 50 min each time, nonadherent cells were resuspended and cultivated in high-glucose DMEM containing 10% FBS, 1% penicillin-streptomycin, and 0.1 mM BrdU for 48 h. The medium was replaced every 48 h.

### AAV9 transfection of cardiomyocytes

After 48 h of culture, NRVMs were transfected with dsAAV9-GFP or dsAAV9-IκBα as previously described [12]. Briefly, cells were first transfected with dsAAV9 (multiplicity of infection, MOI = 5 × 10^6^ vg/cell) in serum-free medium, and then DMEM at an equal volume containing 20% FBS, 2% penicillin-streptomycin and 0.2 mМ BrdU was added to every dish 3 h later. Images showing GFP were captured using a fluorescence inverted microscope (Leica DMI4000B, Weztlar, Germany), and the green fluorescence intensities were analysed using ImageJ software (National Institutes of Health, NY, USA).

### Experimental design and cell grouping

The experiment was designed to explore whether AAV9-delivered IκBα^S32A, S36A^ gene transfection could protect cardiomyocytes against H_2_O_2_-induced apoptosis and autophagy via inhibition of NF-κB activation. Cardiomyocytes were starved with serum-free DMEM for 12 h to ensure cell synchronization before H_2_O_2_ stimulation. The experimental cardiomyocytes were randomly divided into 5 groups as follows: (1) the control group, which contained primary cardiomyocytes cultivated under normal conditions; (2) the H_2_O_2_ control group (H_2_O_2_): the model control group, which contained primary cardiomyocytes subjected to 100 μM H_2_O_2_ alone [12]; (3) the GFP control group (GFP): the vector control group, which contained primary cardiomyocytes transfected with dsAAV9-GFP virus for 5 days before being subjected to 100 μM H_2_O_2_; (4) the IκBα treatment group (IκBα): the treatment group, which contained primary cardiomyocytes transfected with dsAAV9-IκBα virus for 5 days before being subjected to 100 μM H_2_O_2_; and (5) the PDTC treatment group (PDTC): the positive control group, which contained primary cardiomyocytes pretreated with 100 μM PDTC for 60 min before being subjected to 100 μM H_2_O_2_.

### Measurement of cardiomyocyte viability and cytotoxicity

The Cell Counting Kit-8 (CCK-8; Dojindo, Japan) assay was used to assess cell viability. In brief, 2 × 10^4^ cells were seeded into each well of a 96-well plate and transfected with GFP or IκBα for 5 days. After the cells were exposed to H_2_O_2_, 10 μL of CCK-8 stock solution was added to each well, followed by incubation at 37 °C for 2 h. The absorbance at 450 nm was measured with a GO microplate spectrophotometer (Thermo Fisher Scientific). The extent of cell death was determined by quantifying lactate dehydrogenase (LDH) released into the culture supernatant with an LDH kit (Jiancheng Bioengineering Institute, Nanjing, China). Intracellular malondialdehyde (MDA), an indicator of oxidative injury, was also measured using an MDA assay kit (Jiancheng Bioengineering Institute).

### Flow cytometry analysis

Cell apoptosis was measured using PE Annexin V Kit I (BD Biosciences, NJ, USA). Briefly, cells were collected and resuspended in 1× binding buffer. Then, the solution (1 × 10^5^ cells) was supplemented with 5 μL of PE Annexin V and 7-AAD and incubated in the dark for 15 min at room temperature. Apoptotic cells were identified by flow cytometry (Beckman Coulter, CA, USA). All the experiments were performed in triplicate.

### Western blotting analysis

Nuclear and cytoplasmic proteins were extracted following the instructions of a Nuclear and Cytoplasmic Extraction Kit (Thermo Fisher Scientific, USA). Total proteins were extracted with RIPA buffer containing Halt™ Protease and Phosphatase Inhibitor Cocktail. Phosphorylated p65 in the total lysate and the nuclear p65 to cytosolic p65 ratio were both detected to identify activation of the NF-κB signalling pathway [20]. Equal amounts of protein were loaded and separated on precast SDS-PAGE gels (Invitrogen, Grand Island, NY, USA) and transferred to Millipore PVDF membranes. After blocking with 5% skim milk, the membranes were blotted overnight with specific primary antibodies against p65 (1:1000), p-p65 (1:500), IκBα (1:1000), Bax (1:1000), GFP (1:1000), Beclin-1 (1:1000), LC3 II/I (1:1000), Bcl-2 (1:1000), Histone H3 (1:1000), and β-actin (1:1000) at 4 °C, followed by incubation with anti-rabbit HRP secondary antibody (1:5000) at room temperature for 2 h. ECL solution was added to the membranes to visualize signals. β-actin and Histone H3 were regarded as loading controls. Images were captured and analysed by Image Lab 4.0 software (Bio-Rad Laboratories, Hercules, CA, USA).

### Immunofluorescence

Immunofluorescence was employed to identify H_2_O_2_-induced nuclear translocation of the NF-κB p65 subunit in cardiomyocytes. Briefly, 2 × 10^5^ cells were seeded into confocal dishes. After H_2_O_2_ treatment, cardiomyocytes were fixed with 4% paraformaldehyde for 20 min and permeabilized with 0.25% Triton X-100 for 10 min. After blocking with 1% BSA for 1 h, cells were probed overnight with anti-p65 antibody (1:200) at 4 °C and incubated with Alexa Fluor 594-labelled secondary antibody (Invitrogen, 1:200, labelled with red fluorescence) for 2 h at room temperature, followed by 10 min of DAPI staining of nuclei (labelled with blue fluorescence). Signals were detected using a confocal spectral microscope (Leica SP8, Germany).

### Measurement of the mitochondrial membrane potential

JC-1 is an ideal fluorescent probe used to detect the mitochondrial membrane potential (ΔΨm) in cardiomyocytes. Briefly, a 10 nmol/L JC-1 working solution was prepared prior to use, and cardiomyocytes were stained at 37 °C in the dark for 15 min. Cells doubly stained with JC-1 were visible by either green or red fluorescence. Fluorescence images and intensities were obtained using a fluorescence microscope and ImageJ software. Generally, ΔΨm is represented by the red to green fluorescence ratio, which decreases in proportion with the severity of cell injury.

### Statistical analysis

All statistical analyses were performed with SPSS 22.0 (SPSS, Inc., Chicago, IL, USA). Data are presented as the mean ± SEM. Multiple comparisons were carried out using one-way analysis of variance (ANOVA) followed by Bonferroni’s post hoc test. A value of *P* < 0.05 indicated statistical significance.

## Results

### H_2_O_2_-induced activation of NF-κB in NRVMs

**Fig. 1:**
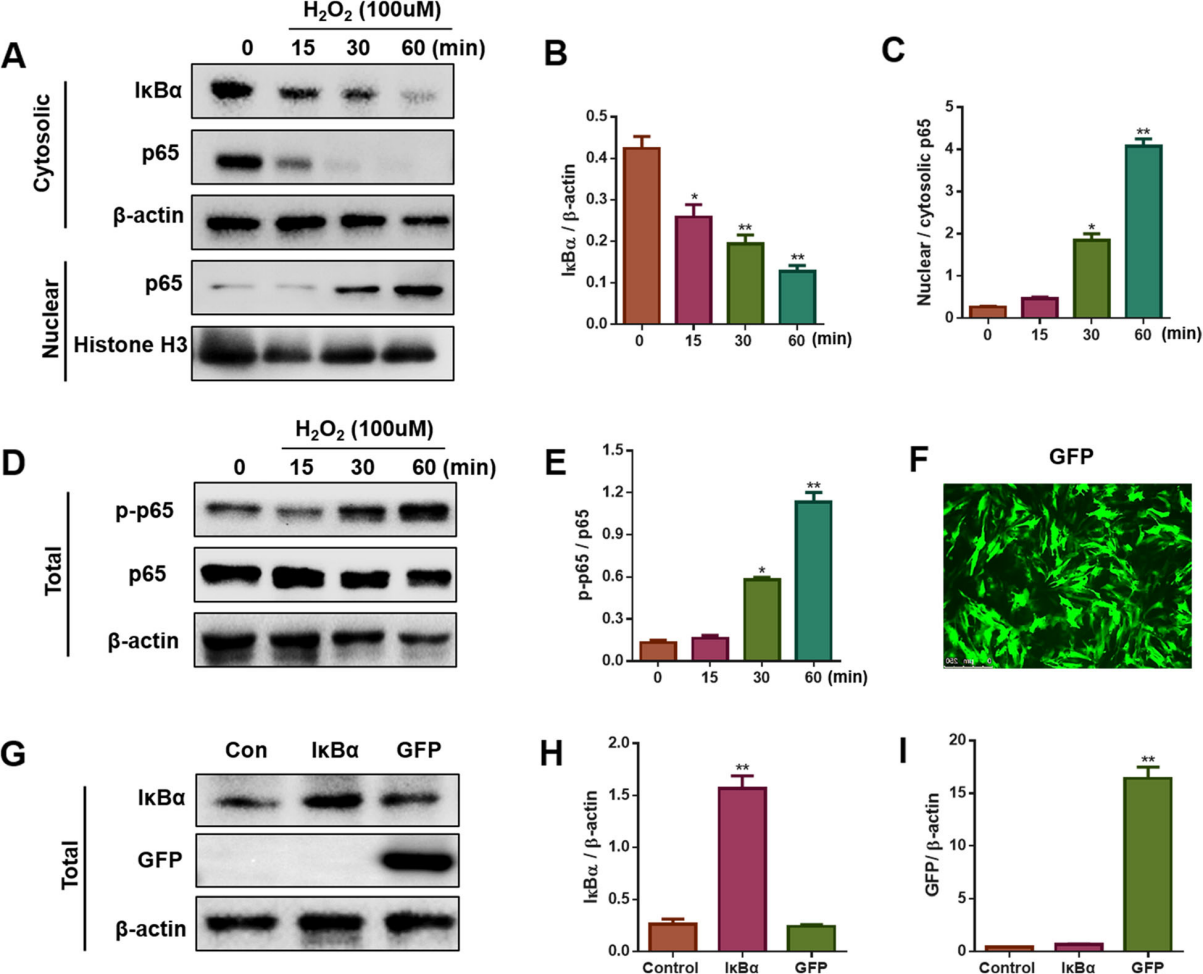
Effects of H_2_O_2_ and AAV9 vectors on NRVMs. **a-c** Western blotting and quantified cytosolic or nuclear protein levels of IκBα and p65 in NRVMs exposed to 100 μM H_2_O_2_ for 0, 15, 30, and 60 min (*n* = 3, *P < 0.05 and **P < 0.01 vs. H_2_O_2_ (0 min)). **d-e** Western blotting and quantified total protein levels of p-p65 and p65 in NRVMs subjected to 100 μM H_2_O_2_ for 0, 15, 30, 60 min (n = 3, *P < 0.05 and **P < 0.01 vs. H_2_O_2_ (0 min)). **f** The green fluorescence intensity of GFP in NRVMs (scale bar: 250 μm). **g-i** Western blotting to detect the protein expression of IκBα and GFP. (n = 3, ** *P* < 0.01 vs. Control)

The results indicated that H_2_O_2_ elicited time-dependent IκBα degradation and p65 translocation after the NRVMs were incubated with 100 μM H_2_O_2_ for different durations (0, 15, 30, and 60 min). (Fig. 1a-c). The ratio of nuclear p65 to cytosolic p65 peaked at 60 min. Consistent with the nuclear translocation of p65, the level of p-p65/p65 increased following H_2_O_2_ stimulation with the incubation time (Fig. 1d and e) and was highest at 60 min. Thus, treatment with 100 μM H_2_O_2_ for 60 min was used in the following experiments.

### Efficiency of IκBα transfection in NRVMs

As shown in Fig. 1f, the green fluorescence signal was robust, and the dsAAV9-GFP transfection efficiency in NRVMs reached more than 70%. Western blotting analysis showed that the GFP protein was more highly expressed in the GFP group than in the other groups, while the IκBα protein level was significantly elevated in the IκBα group compared with the control and GFP groups (Fig. 1g-i).

### IκBα protected cardiomyocytes from H_2_O_2_-induced apoptosis

**Fig. 2:**
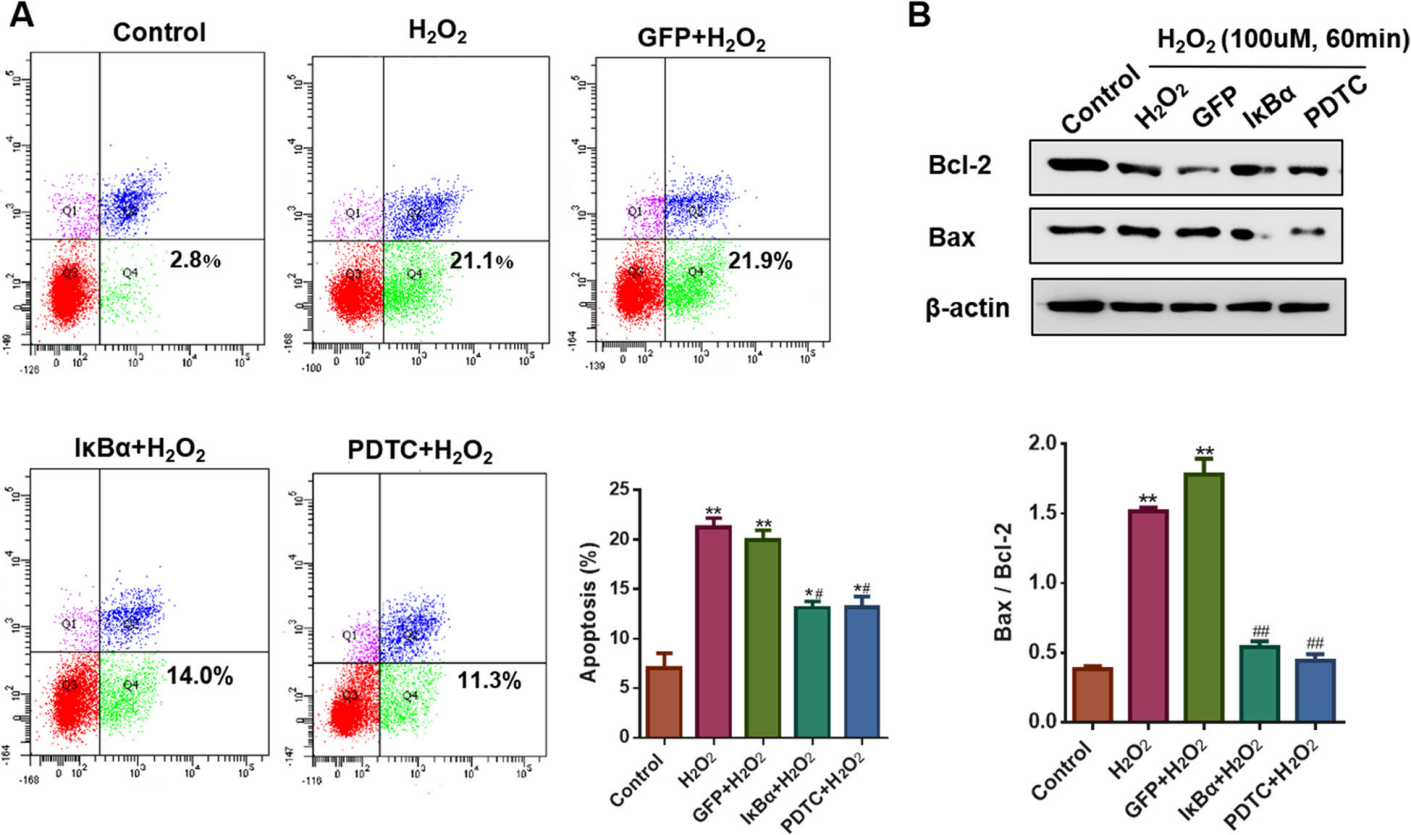
IκBα reduced cell apoptosis in NRVMs exposed to H_2_O_2_. **a** Flow cytometry indicated that IκBα or PDTC attenuated cell apoptosis in NRVMs exposed to H_2_O_2_ (n = 3, * *P* < 0.05 and ** *P* < 0.01 vs. Control, ^#^*P* < 0.05 vs. H_2_O_2_ group. **b** Western blotting findings indicated that IκBα or PDTC reduced the ratio of Bax/Bcl-2 in NRVMs subjected to H_2_O_2_ (n = 3, ** *P* < 0.01 vs. Control, ^##^*P* < 0.01 vs. H_2_O_2_ group)

The proportion of apoptotic cells in the control group was 7.0 ± 1.5%. After treatment with 100 μM H_2_O_2_, the apoptotic rates of cardiomyocytes in the H_2_O_2_ group and GFP group increased to 21.20 ± 0.95% and 19.97 ± 0.97%, respectively, which were decreased by IκBα or PDTC pretreatment (Fig. 2a). Indeed, compared with the levels in the control group, the anti-apoptotic protein Bcl-2 was downregulated, but the pro-apoptotic protein Bax was upregulated in NRVMs exposed to H_2_O_2_, leading to a higher Bax/Bcl-2 ratio, but this effect was completely abolished by pretreatment with IκBα or PDTC (Fig. 2b).

### IκBα protected cardiomyocytes from H_2_O_2_-induced cell injury

**Fig. 3:**
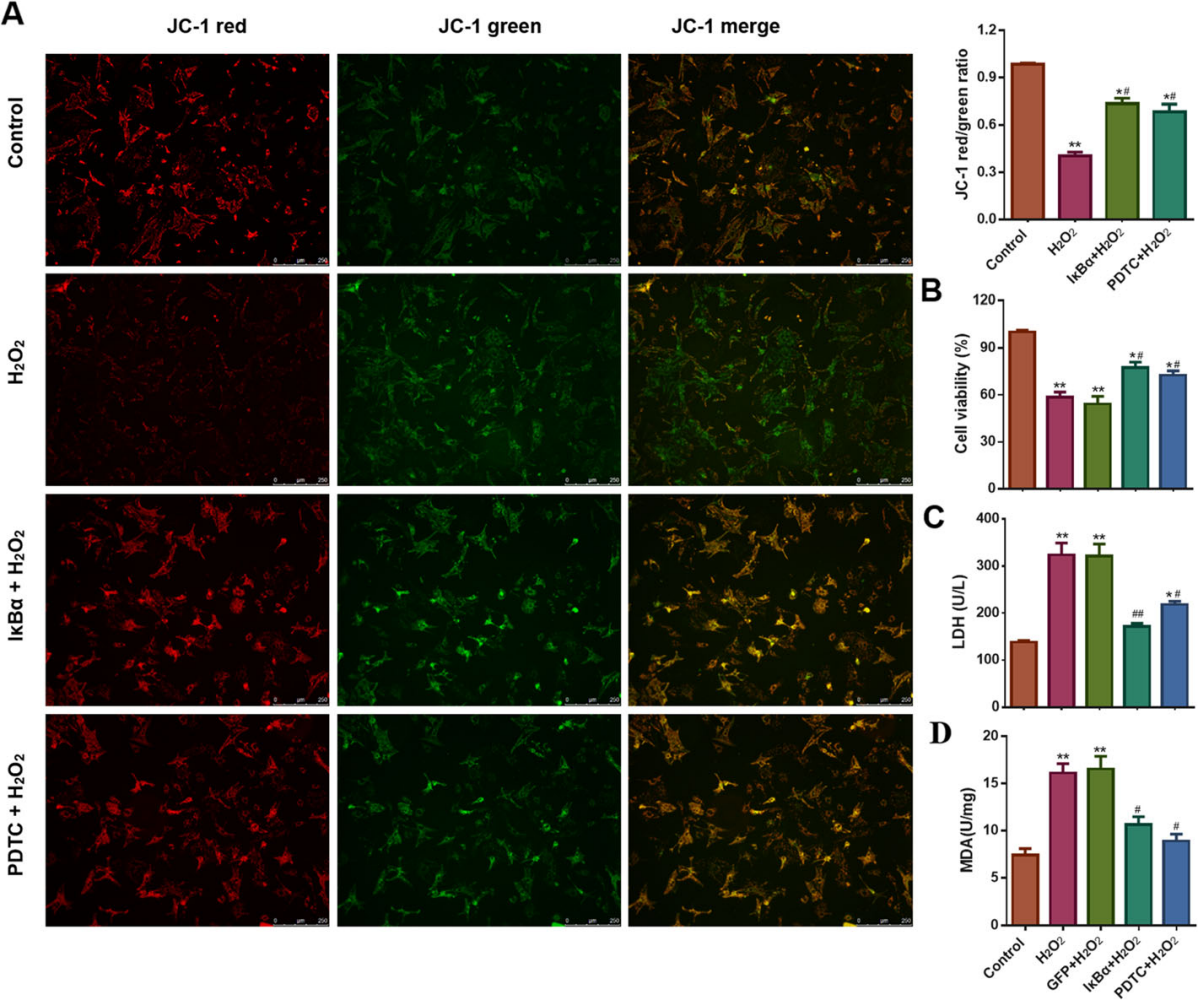
IκBα reduced cell injury in NRVMs exposed to H_2_O_2_. **a** Representative images of JC-1 staining in different groups (scale bar: 250 μm). The results indicated that IκBα or PDTC elevated the ratio of red to green fluorescence intensity (n = 3, * *P* < 0.05 and ** *P* < 0.01 vs. Control, ^#^*P* < 0.05 vs. H_2_O_2_ group). **b** CCK-8 results demonstrated that IκBα or PDTC increased the cell viability of NRVMs stimulated by H_2_O_2_ (n = 3, * *P* < 0.05 and ** *P* < 0.01 vs. Control, ^#^*P* < 0.05 vs. H_2_O_2_ group). **c** IκBα or PDTC reduced the levels of supernatant LDH in NRVMs subjected to H_2_O_2_ (n = 3, * *P* < 0.05 and ** *P* < 0.01 vs. Control, ^#^*P* < 0.05 and ^##^*P* < 0.01 vs. H_2_O_2_ group). **d** IκBα or PDTC decreased the content of intracellular MDA in H_2_O_2_-treated NRVMs (n = 3, ** *P* < 0.01 vs. Control, ^#^*P* < 0.05 vs. H_2_O_2_ group)

Compared to that in the control group, ΔΨm was significantly decreased in the H_2_O_2_ control group, but this decrease was rescued by IκBα or PDTC treatment (Fig. 3a). Additionally, H_2_O_2_ treatment significantly decreased cell viability but elevated supernatant LDH and intracellular MDA levels; however, these changes were reversed by IκBα or PDTC treatment (Fig. 3b-d).

### IκBα suppressed H_2_O_2_-induced NF-κB activation and autophagy in NRVMs

**Fig. 4:**
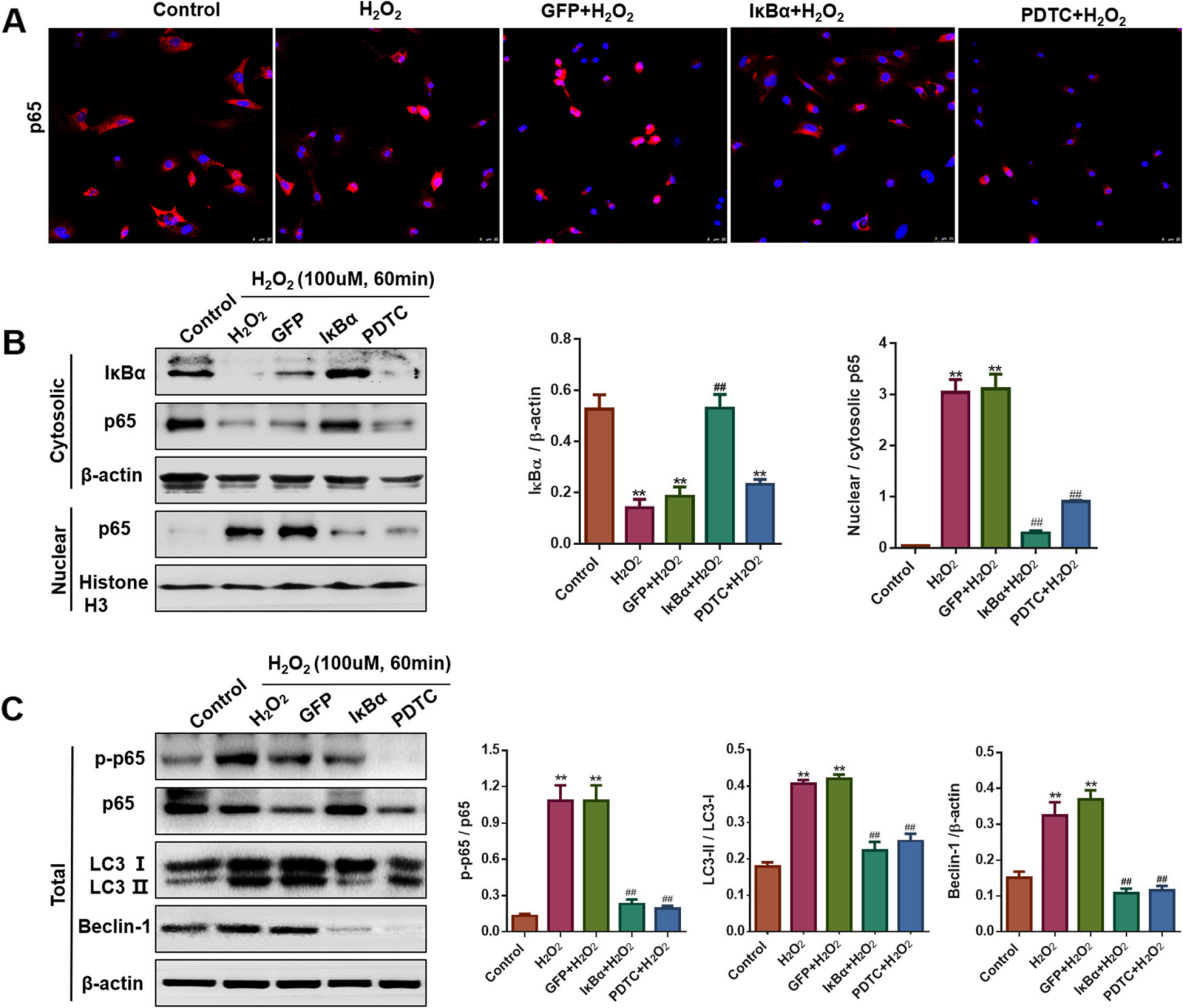
IκBα suppressed H_2_O_2_-induced NF-κB activation and autophagy in NRVMs. **a** The distribution of NF-κB p65 (red fluorescence) was detected using immunofluorescence (scale bar: 25 μm). The results showed that IκBα or PDTC inhibited H_2_O_2_-induced p65 nuclear translocation. **b** Western blotting and quantified cytosolic or nuclear protein levels of IκBα and p65 in NRVMs exposed to 100 μM H_2_O_2_. The results showed that IκBα transduction maintained the cytosolic IκBα level and suppressed p65 nuclear translocation (n = 3, ** *P* < 0.01 vs. Control, ^##^*P* < 0.01 vs. H_2_O_2_ group). **c** Western blotting and quantified total protein levels of p-p65/p65, Beclin-1 and LC3-II/LC3-I in NRVMs exposed to 100 μM H_2_O_2_. The findings showed that IκBα or PDTC decreased the p-p65/p65 ratio, Beclin-1 expression and the LC3-II/LC3-I ratio after H_2_O_2_ stimulation. (n = 3, ** *P* < 0.01 vs. Control, ^##^*P* < 0.01 vs. H_2_O_2_ group)

Compared with the control group, H_2_O_2_ treatment significantly elicited p65 translocation, and these changes were successfully reversed by IκBα or PDTC pretreatment (Fig. 4a and b). Consistently, H_2_O_2_ increased p-p65/p65 ratio in NRVMs, but IκBα or PDTC dramatically downregulated the H_2_O_2_-induced expression of p-p65. Meanwhile, Beclin-1 and the LC3-II/LC3-I ratio, the autophagy-associated markers, were markedly upregulated in NRVMs exposed to H_2_O_2_, whereas these effects were inhibited by IκBα or PDTC treatment (Fig. 4c).

## Discussion

This study shows that IκBα degradation and NF-κB activation occurred in a time-dependent manner in NRVMs subjected to H_2_O_2_. Cells treated with H_2_O_2_ showed reductions in cell viability and ΔΨm but elevations in LDH and MDA levels, apoptosis and autophagy. IκBα transfection or PDTC pretreatment ameliorated H_2_O_2_-induced cell injury by inhibiting NF-κB activation.

I/R injury severely attenuates the benefit of revascularization after AMI and has therefore become an important focus of cardiovascular research [2]. The inflammatory response induced by AMI is essential for heart repair, but excessive generation of ROS and inflammation following reperfusion therapy exacerbate heart damage [21].

The NF-κB signalling pathway plays key roles in the inflammatory response, oxidative stress, apoptosis, and autophagy in the heart [8]. Phosphorylation and nuclear translocation of the p65 subunit are signs of NF-κB activation [20]. Previous studies [22–24] identified that H_2_O_2_ treatment for different durations (30 min-24 h) elicited significant p65 phosphorylation and nuclear translocation in NRVMs. In line with these studies, p65 was time-dependently phosphorylated and translocated from the cytoplasm to the nucleus with IκBα degradation in NRVMs subjected to H_2_O_2_.

However, whether NF-κB activation protects or damages cardiomyocytes remains debatable. An early study demonstrated that activation of NF-κB reduced cell apoptosis in hypoxic cardiomyocytes [25], whereas most recent studies [26, 27] have shown that NF-κB is a pro-apoptotic transcription factor correlated with myocardial injury and that blockade of NF-κB activity prevents myocardial apoptosis. Gray et al. [22] recently reported that ROS generated by ischaemia-reperfusion could rapidly activate calmodulin kinase II (CaMKII), which increased cell injury by inducing IκBα degradation and nuclear p65 accumulation in NRVMs exposed to H_2_O_2_. Importantly, knockout of the CaMKIIδ gene significantly attenuated myocardial infarct size by inhibiting IκBα degradation and NF-κB activation. All these findings show that NF-κB activation deteriorates the heart in I/R injury.

Herein, we hypothesized that direct overexpression of IκBα to prevent NF-κB activation may have a good protective effect in cardiomyocytes. Then, dsAAV9-IκBα^Ser 32A,36A^ was designed to prevent IκBα degradation due to its phosphorylation at the Ser 32 and Ser 36 sites and successfully transfected into cardiomyocytes. Western blotting and immunofluorescence demonstrated that IκBα transfection successfully maintained cytoplasmic IκBα levels and suppressed p65 phosphorylation and translocation in NRVMs exposed to H_2_O_2._ Additionally, IκBα elevated cell viability, decreased LDH and MDA levels, and attenuated apoptosis, implying the protective role of IκBα in H_2_O_2_-induced cell injury in NRVMs. The mechanisms may account for the role of NF-κB in mediating the expression of various proteins that promote or inhibit apoptosis. Notably, NF-κB regulates the expression of certain anti-apoptotic genes, such as Bcl-2 [28], and an increased Bcl-2/Bax ratio decreases cell apoptosis. In this study, treatment with IκBα or PDTC significantly elevated the Bcl-2/Bax ratio in NRVMs subjected to H_2_O_2_. These data indicate that IκBα protects NRVMs against H_2_O_2_-induced apoptosis by increasing the Bcl-2/Bax ratio.

Opening of the mitochondrial permeability transition pore (MPTP) in the first few minutes of reperfusion leads to ΔΨm loss and is responsible for necrotic and apoptotic cell death processes, contributing differentially to myocardial infarct size [29]. Importantly, inhibition of the opening of the MPTP attenuated I/R injury. Thus, ΔΨm loss reflects mitochondrial dysfunction, indicates early-stage apoptosis and is a critical determinant of I/R injury [30]. A previous study demonstrated that H_2_O_2_ induced a significant decrease in ΔΨm [12]. In this study, H_2_O_2_ treatment attenuated ΔΨm and enhanced Bax expression in NRVMs, and these effects were reversed by pretreatment with IκBα or PDTC. NF-κB is involved in the regulation of mitochondrial dysfunction [31], and Bax antagonizes the anti-apoptotic ability of Bcl-2 and simultaneously promotes permeability of the mitochondrial outer membrane and reduces the level of ΔΨm [32]. The results herein suggest that IκBα decreases cell injury and apoptosis by inhibiting NF-κB activation and Bax expression, ultimately elevating ΔΨm after H_2_O_2_ stimulation.

Autophagy, a cellular process of lysosome-mediated degradation of cytoplasmic

components or damaged organelles, is thought to be an adaptive response and protective for cell survival [10]. However, autophagy causes a redox effect in cardiomyocytes when exposed to different stimuli. Evidence supports the benefit of autophagy to cardiomyocytes during myocardial ischaemia through its improvement of myocardial energy metabolism and organelle recycling [33], but excessive autophagy causes lethal damage to cells during cardiac I/R injury [21], which is mediated in part by upregulation of Beclin-1 expression [34].

However, the communication between autophagy and NF-κB is bidirectional. Autophagy is required for activation of NF-κB [13]; in turn, NF-κB further increases autophagosome maturation by upregulating Beclin-1 and LC3-II expression in I/R injury [35]. Importantly, PDTC attenuates Beclin-1 expression and the formation of autophagosomes by suppressing I/R injury-induced NF-κB activation [16]. In accordance with these findings, treatment of NRVMs with H_2_O_2_ induced p65 phosphorylation and translocation, enhanced Beclin-1 expression, and increased the LC3-II/LC3-I ratio, and these effects were rescued by IκBα transfection or PDTC treatment. These results imply that IκBα can protect cardiomyocytes by inhibiting H_2_O_2_-induced autophagy. In addition, Bcl-2 can bind Beclin-1 to inhibit autophagy [36]. This study also demonstrates that IκBα transfection elevated the expression of Bcl-2, which may disturb the function of Beclin-1 and thus further inhibit H_2_O_2_-induced autophagy, implying cross-talk between apoptosis and autophagy.

### Study strengths and limitations

The present study has several strengths. To the best of our knowledge, this study is the first to report the cardioprotective effects of IκBα transfection through inhibition of H_2_O_2_-induced apoptosis and autophagy. Moreover, AAV9 vectors, which can be successfully transfected into cardiomyocytes, may be used as an effective tool for gene therapy for AMI. This study also has some limitations. The present findings were derived from neonatal cardiomyocytes in vitro and may differ from findings in animal experiments due to the complicated features of the in vivo environment. Further animal studies should be conducted to confirm the cardioprotective effects of IκBα against I/R injury.

## Conclusions

The findings of this study show that pretreatment with dsAAV9-IκBα or PDTC protected NRVMs from H_2_O_2_-induced apoptosis, autophagy, mitochondrial dysfunction, and oxidative damage by restraining the NF-κB signalling pathway, suggesting that IκBα transfection can protect cardiomyocytes against cardiac oxidative damage. Thus, AAV9 vectors, as high-efficiency gene carriers to heart, may be used to carry the IκBα gene to protect the heart through targeted inhibition of myocardial NF-κB in future preclinical or clinical studies, which may provide a promising gene therapy for preventing cardiac I/R injury.

## Data Availability

All data generated or analysed during this study are included in this article.

## Abbreviations

NF-κB: Nuclear factor kappa B
IκBα: Inhibitor of kappa B alpha
NRVMS: Neonatal rat ventricular cardiomyocytes
I/R: Ischaemia/reperfusion
AMI: Acute myocardial infarction
H_2_O_2_: Hydrogen peroxide
GFP: Green fluorescent protein
PDTC: Pyrrolidine dithiocarbamate
CCK-8: Cell counting kit-8
LDH: Lactate dehydrogenase
MDA: Malondialdehyde
MPTP: Mitochondrial permeability transition pore
ΔΨm: Mitochondrial membrane potential
ROS: Reactive oxygen species
